# Phenomics-assisted sparse testing for potato breeding

**DOI:** 10.1038/s41598-026-59202-6

**Published:** 2026-07-14

**Authors:** Alexandre Hild Aono, Aakash Chawade

**Affiliations:** Department of Plant Breeding, Swedish University of Agricultural Sciences (SLU), SE-230 53 Alnarp, Sweden

**Keywords:** Genomic selection, Genotype-by-environment interaction, High-throughput phenotyping, Multi-environment regression, *Solanum tuberosum*, Computational biology and bioinformatics, Genetics, Plant sciences

## Abstract

In recent decades, global weather patterns have shifted dramatically, introducing greater unpredictability into agriculture. A major challenge in plant breeding is developing selection strategies that remain accurate under such uncertainty. Sparse testing is a well-established approach to increase the number of genotypes evaluated in field trials while keeping costs manageable. However, incorporating image-based data into sparse testing remains challenging. We developed a strategy to integrate high-throughput phenotyping data into sparse testing in potato breeding to improve predictive performance in multi-environment trials. Our approach involved constructing an environmental kernel derived from the covariance matrix of image-based data. We assessed the predictive performance of several regression models under sparse testing, including those based on genomic or phenomic data alone and in combination. Models using only the proposed environmental kernel achieved predictive accuracies comparable to, or exceeding, those of genomic prediction models in various sparse testing scenarios. The best results were observed for tuber yield, a key trait in potato breeding. These findings highlight the potential of image-based environmental kernels to improve the efficiency and accuracy of sparse testing. This approach is cost-effective and scalable, particularly useful for breeding programs with limited resources.

## Introduction

Plant breeding has its roots in antiquity and has evolved from a practical art into a sophisticated science focused on improving plant traits through a wide array of strategies and technologies. These include not only traditional phenotypic selection but also modern techniques incorporating biotechnology, robotics, and advanced data analytics^1^. Over time, breeding programs for various crops have successfully released high-yielding cultivars. However, one of the most pressing current challenges lies in developing effective selection strategies under the uncertainty imposed by climate change^2^.

In recent decades, global weather patterns have shifted dramatically due to climate change. Rising temperatures, fluctuating sea levels, increased frequency of droughts, and other climatic disruptions have intensified the urgency for developing resilient cultivars^3^. Selecting genotypes based on performance in a particular year and location may not translate to superior performance in subsequent seasons, due to unpredictable environmental variability^4^. Consequently, modern statistical modeling approaches must incorporate environmental variation to more accurately predict plant performance across time and space.

Among the most impactful tools in modern breeding is genomic selection (GS), which has markedly increased genetic gains across multiple crops^5^. GS enables early selection based on genomic estimated breeding values (GEBVs), thereby shortening the breeding cycle and improving selection accuracy^6^. Nonetheless, one of the main challenges in GS lies in predicting the performance of new genotypes in untested environments. Due to substantial year-to-year climate variability and strong genotype-by-environment interactions, model predictions may become unreliable, potentially leading to suboptimal selection decisions^7^.

To address this challenge, genome-enabled sparse testing has been suggested as a promising strategy for implementing GS more efficiently^8,9^. This approach reduces the number of genotypes phenotyped in each environment while maintaining the overall number of environments or years tested. Sparse testing can enhance prediction accuracy while significantly increasing the evaluation capacity^10^.

Another cost-effective alternative to conventional phenotyping is high-throughput phenotyping (HTP), which leverages automation technologies to streamline data collection. The use of unmanned aerial vehicles (UAVs), for example, enables the rapid collection of aerial imagery to extract digital phenotypes across multiple environments^11^. These technologies support not only real-time crop monitoring but also the development of predictive models to estimate trait performance throughout breeding cycles^12^.

Recent studies have compared the genetic gains and costs associated with GS, HTP, and their integration^13,14^. While sparse testing is considered a highly promising strategy for maximizing genetic gain through GS, its applicability using HTP data has not been thoroughly assessed. A fundamental limitation is that HTP-derived phenotypes, being collected in field trials, cannot be obtained for individuals excluded from phenotyping, which contrasts with GS, where genotyping can be done at the seedling stage regardless of field performance^10^.

Potato (*Solanum tuberosum* L.), one of the most important crops for global food security, continues to face slow breeding progress due to its low multiplication rate and high disease susceptibility^15^. These constraints underscore the need for innovative breeding approaches. Potato’s self-compatible, polysomic tetraploid genome (2n = 4x = 48) presents additional complexity for the development of GS models^16^. Furthermore, as a tuber crop, accurately predicting belowground traits such as tuber yield and quality using UAV-derived aboveground phenotypes remains a significant challenge^17^. In this context, sparse testing with HTP offers a potentially powerful approach to enhance genetic gains in potato breeding programs.

In this study, we assessed the feasibility of implementing sparse testing using HTP-derived data in a diverse panel of potato breeding clones and cultivars, evaluated across three environments. To predict key agronomic traits, such as total tuber weight, tuber size distribution, and starch content, we compared multi-environment genomic prediction models with a newly developed multi-environment phenomic prediction approach. Our findings offer insights into the potential of combining phenomics and sparse testing to accelerate genetic gains in potato breeding under climate-resilient selection frameworks.

## Material and methods

### Plant material and conventional phenotyping

In this study, we evaluated a potato population composed of 256 breeding clones and cultivars, fully listed in Supplementary Table S1. The plant material employed in the present study are in compilance with institutional, national, and international guidelines and legislation.

Field trials were carried out using incomplete block designs at two rural sites in southern Sweden: Helgegården (56$^{\circ }$00$^{\prime }$56$^{\prime \prime }$N/14$^{\circ }$03$^{\prime }$50$^{\prime \prime }$E) and Mosslunda (55$^{\circ }$58$^{\prime }$04$^{\prime \prime }$N/14$^{\circ }$06$^{\prime }$51$^{\prime \prime }$E). At Helgegården, trials took place in 2020 with 169 genotypes arranged in a 13 $\times$ 13 simple lattice design and evaluated in two replications. In 2021, trials at Helgegården included the full set of 256 genotypes, arranged in a 16 $\times$ 16 simple lattice design with two replications. At Mosslunda, a single trial was conducted in 2021 using the full set of genotypes and the same lattice design of Helgegården in 2021. Each location-year combination was treated as a unique environment for subsequent analysis.

The local growing season typically extends from late May through early September, lasting roughly 3.5 to 4 months. To capture environmental variation across sites and seasons, we obtained daily weather and elevation data using the EnvRtype R package v1.1.2^18^. Environmental covariates considered included: (i) temperature at 2 m ($^{\circ }$C); (ii) maximum temperature at 2 m ($^{\circ }$C); (iii) minimum temperature at 2 m ($^{\circ }$C); (iv) dew/frost point at 2 m (m/s); (v) downward thermal infrared (longwave) radiative flux; (vi) all-sky insolation on a horizontal surface; (vii) all-sky downward direct normal shortwave irradiance; (viii) all-sky photosynthetically active radiation (PAR); (ix) all-sky ultraviolet A irradiance (315–400 nm); (x) all-sky ultraviolet B irradiance (280–315 nm); (xi) precipitation (mm); (xii) evapotranspiration energy flux at the surface; (xiii) specific humidity at 2 m (kg water/kg total air); (xiv) relative humidity at 2 m; (xv) root-zone soil wetness (0–100 cm); (xvi) surface soil wetness (0–5 cm); (xvii) wind speed at 2 m (m/s); (xviii) precipitation minus evapotranspiration; (xix) vapor pressure deficit (kPa); (xx) photoperiod (h); (xxi) top-of-atmosphere radiation; (xxii) number of theoretical sunny hours per day under clear skies; (xxiii) temperature–humidity index 1^19^; (xxiv) temperature–humidity index 2^20^; and (xxv) the ratio of PAR to temperature.

Despite the close proximity of the trial sites, they belong to different 0.5$^{\circ }$ grid cells in the NASA POWER database^21^, which may slightly differentiate the environmental data retrieved. To analyze seasonal patterns, monthly averages for each covariate were calculated for every environment. These values were visualized using heatmaps created with the pheatmap package v1.0.12^22^ in R v4.1.2^23^. Pairwise Pearson correlations were also computed using the monthly means to examine similarities between environments. Additionally, daily temperature curves for 2020 and 2021 were plotted using ggplot2 v3.5.1^24^ to depict temporal trends.

The set of conventional traits evaluated comprised total tuber weight (kg); tuber weight (kg) within defined size ranges (<40 mm, 40–50 mm, 50–60 mm, and >60 mm); and tuber starch content (%).

### High-throughput phenotyping

For each field trial, we also evaluated image-based traits obtained from drone imagery collected at multiple time points in each environment (Table 1). All flights were conducted at an altitude of 30 meters. The UAV used was a DJI Mavic 2 Pro UAV, equipped with a 20 MP RGB camera.

**Table 1:** Time points for aerial image acquisition in each environment.

Location	Year	June	July	August	September
Helgegården	2020	–	8, 22, 31	–	2, 10, 16
Helgegården	2021	–	7, 20	4, 24	9
Mosslunda	2021	28	7, 20	4, 24	–

Drone image processing was carried out using the software Pysel (Phenoyard AB, Sweden), which included the following steps for each flight-environment combination: (i) orthomosaic generation; (ii) definition of experimental plots ; and (iii) extraction of plant measurements and vegetation indices, including: visible atmospherically resistant index (VARI), green-red ratio index (GRRI), canopy reflectance (CR), coverage, plant height, and leaf chlorophyll content.

For the vegetation indices VARI and GRRI, pixel-wise values were averaged at the plot level for each time point. For all plant measurements and vegetation indices, the temporal profiles were combined into image-based traits by calculating the area under the curve (AUC) using the trapezoidal rule:

1 $$\begin{aligned} \textrm{AUC}=\sum _{i=1}^{n-1}{\frac{\left( T(t_i)+T(t_{i+1})\right) }{2}}\times \left( t_{i+1}-t_i\right) , \end{aligned}$$

where $T(t_i)$ and $T(t_{i+1})$ are the index values at successive time points $t_i$ and $t_{i+1}$, respectively. We used these AUC values to represent crop-growth dynamics instead of single-point evaluations, which can be sensitive to environmental fluctuations and difficult to replicate.

### Phenotypic data analyses

The initial step in evaluating the phenotypic data involved generating density plots for the raw measurements of both conventional (total tuber weight (kg), tuber weight (kg) within defined size ranges, and tuber starch content) and image-based traits (VARI, GRRI, CR, coverage, plant height, and leaf chlorophyll content). This was performed using the R statistical software v4.1.2^23^ and the ggplot2 v3.5.1 package^24^. In addition, a principal component analysis (PCA) was conducted on the raw values of the image-based traits. Subsequently, for each conventional and image-based trait, we modeled the following linear mixed-effects model using the ASReml-R package v4.2.0.355^25^:

2 $$\begin{aligned} \underline{y}_{ijklm}=\mu +e_j+r_{jk}+\underline{p}_{jkl}+\underline{c}_{jkm}+\underline{g}_i+\underline{ge}_{ij}+\underline{\epsilon }_{ijklm}, \end{aligned}$$

where $\underline{y}_{ijklm}$ is the observed trait value for the *i*-th genotype in the *k*-th replicate of the *j*-th environment, located in the *l*-th row and *m*-th column; $\mu$ is the fixed overall population mean; $e_j$ is the fixed effect of the *j*-th environment; $r_{jk}$ is the fixed effect of the *k*-th replicate within the *j*-th environment; $\underline{p}_{jkl}$ is the random effect of the *l*-th row within the *k*-th replicate and *j*-th environment ($\underline{p}_{jkl}\sim \textrm{N}(0,\sigma _p^2)$, with $\sigma _p^2$ representing the row-by-replication-by-environment variance); $\underline{c}_{jkm}$ is the random effect of the *m*-th column within the *k*-th replicate and *j*-th environment ($\underline{c}_{jkm}\sim \textrm{N}(0,\sigma _c^2)$, with $\sigma _c^2$ representing the column-by-replication-by-environment variance); $\underline{g}_i$ is the random effect of the *i*-th genotype ($\underline{g}_i\sim \textrm{N}(0,\sigma _g^2)$, with $\sigma _g^2$ representing the genetic variance); $\underline{ge}_{ij}$ is the random effect of the interaction between the *i*-th genotype and the *j*-th environment ($\underline{ge}_{ij}\sim \textrm{N}(0,\sigma _{ge}^2)$, with $\sigma _{ge}^2$ representing the genotype-by-environment interaction variance); and $\underline{\epsilon }_{ijklm}$ is the residual error ($\underline{\epsilon }_{ijklm}\sim \textrm{N}(0,\sigma _\epsilon ^2)$, where $\sigma _\epsilon ^2$ is the residual variance). All random terms were underlined and the random effects were assumed to be independently and identically distributed. The block factors in the experimental design corresponded to the row and column effects.

For estimating variance components, we used the restricted maximum likelihood (REML) approach. The significance of fixed effects were assessed using Wald F-tests, while random effects were tested using likelihood ratio tests (LRTs) through analyses of deviance (ANODEV). We estimated broad-sense heritability ($h^2$) as^26^:

3 $$\begin{aligned} h^2=\frac{\sigma ^2_g}{\sigma ^2_g+\frac{\sigma ^2_p}{r\times m}+\frac{\sigma ^2_c}{r\times m}+\frac{\sigma ^2_{ge}}{m}+\frac{\sigma ^2_{\epsilon }}{r\times m}}, \end{aligned}$$

where *r* is the number of replicates and *m* the number of environments. The terms $\sigma _g^2$, $\sigma _p^2$, $\sigma _c^2$, $\sigma _{ge}^2$, and $\sigma _\epsilon ^2$ are the estimated variance components. We also computed Cullis heritability ($h_C^2$)^27^ to evaluate the precision of genotypic predictions:

4 $$\begin{aligned} h_C^2=1-\frac{\textrm{PEV}}{2\sigma ^2_g}, \end{aligned}$$

where $\textrm{PEV}$ is the prediction error variance (average variance of the genotypic best linear unbiased predictors, BLUPs) and $\sigma ^2_g$ the estimated genetic variance component.

To evaluate genotype-specific variance patterns across environments, we re-fitted the mixed model excluding the main genotype effect ($\boldsymbol{g}$), thereby focusing on predicting genotype-by-environment interaction ($\boldsymbol{ge}$) BLUPs. To better account for environmental variability, we modeled both the interaction ($\boldsymbol{ge}$) and the residuals ($\boldsymbol{\epsilon }$) using separable variance-covariance structures. Specifically, we assumed $\boldsymbol{G_E}=\boldsymbol{E}\otimes \boldsymbol{I}_n$ ($\boldsymbol{ge}\sim \textrm{MVN}(0,\boldsymbol{G_E})$) and $\boldsymbol{R_E}=\boldsymbol{E}\otimes \boldsymbol{I}_{n_2}$ ($\boldsymbol{\epsilon }\sim \textrm{MVN}(0,\boldsymbol{R_E})$). Here, $\boldsymbol{E}$ is the environment ($m\times m$) variance-covariance matrix, $\boldsymbol{I}_n$ and $\boldsymbol{I}_{n_2}$ are identity matrices corresponding to genotypes and residuals (number of plots), respectively. We evaluated the following structures for the $\boldsymbol{E}$ matrix: (i) identity; (ii) diagonal; (iii) compound symmetry; (iv) heterogeneous compound symmetry; (v) first-order autoregressive; (vi) heterogeneous first-order autoregressive; and (vii) unstructured.

Model comparisons were performed using Akaike’s information criterion (AIC)^28^ and Bayesian information criterion (BIC)^29^. Based on the lowest AIC and BIC values, the heterogeneous first-order autoregressive structure was selected for both conventional and image-based traits. BLUPs obtained from models incorporating this structure were used to compute pairwise trait correlations. All analyses were performed in R version 4.1.2^23^, with visualizations generated using the ggplot2 v3.5.1^24^ and GGally v2.1.2^30^ packages.

For use in prediction models, we re-fitted the models by excluding the main genotype effect ($\boldsymbol{g}$and treating the genotype-by-environment interaction term ($\boldsymbol{ge}$) as a fixed effect. Best linear unbiased estimates (BLUEs) for $\boldsymbol{ge}$ were estimated and retained for downstream analyses. For image-based traits, a min–max normalization was applied using the following formula:

5 $$\begin{aligned} f(v)=\frac{v-\textrm{min}(v)}{\textrm{max}(v)-\textrm{min}(v)}, \end{aligned}$$

where each value *v* was scaled to the range [0, 1] (*f*(*v*)), based on the minimum ($\textrm{min}(v)$) and maximum ($\textrm{max}(v)$) values across the trait.

PCA and Pearson correlation analyses were performed using R statistical software v4.1.2^23^, with visualizations generated using the ggplot2 v3.5.1^24^ and GGally v2.1.2^30^ packages. For the image-based traits, we also constructed a phenomic relationship matrix using R v4.1.2^23^ and the pheatmap package v1.0.12^22^. This matrix quantifies pairwise differences between individuals based on Euclidean distances computed from the image-based trait BLUEs.

### Genotyping

Genotyping data were obtained using the DArTag approach, conducted by Diversity Arrays Technology Pty Ltd (ACT, Australia), based on leaf DNA samples processed by Intertek ScanBi Diagnostics (Alnarp, Sweden). The single nucleotide polymorphism (SNP) assay included 2,500 SNPs derived from prior genotyping projects and selected to meet a minimum allele frequency (MAF) of 1% in genotypes from the breeding programs of the International Potato Center (CIP, Lima, Peru) and the University of Wisconsin (United States).

A closely related Nordic population was investigated by^31^, who demonstrated in^32^ that a reduced SNP set was sufficient to characterize the genetic diversity of Nordic potato genotypes. Building on these findings, several genomic prediction studies were subsequently conducted using the same population analyzed in our study, further supporting the effectiveness and predictive accuracy of this SNP panel^33–35^.

Three individuals were excluded due to excessive missing genotype data: the chip potato breeding clone 97 (Swedish University of Agricultural Sciences), and the cultivars Leyla (released in Germany in 1988) and Red Lady (released in Germany in 2004). The remaining individuals had no missing values and were retained for genomic prediction analyses. SNPs were coded as tetraploid allele dosages, where 0 represents the reference homozygous class, 4 the alternative homozygous class, and 1, 2, and 3 the heterozygous classes.

To assess population structure, PCA was performed using R v4.1.2^23^, and the resulting principal components were visualized with the ggplot2 v3.5.1 package^24^. In addition, we evaluated the genomic relationship matrix $\boldsymbol{G}$, which was visualized using the pheatmap package v1.0.12^22^. The matrix $\boldsymbol{G}$ was computed using the centered SNP matrix $\boldsymbol{W}$ across *n* genotypes $\boldsymbol{G}=\frac{\boldsymbol{WW^T}}{\textrm{tr}(\boldsymbol{WW^T})/n}$ using the R package AGHmatrix v2.1.0^36^. A dissimilarity matrix derived from $\boldsymbol{G}$ was used as input for hierarchical clustering based on Ward’s minimum variance method^37^. Clusters of individuals were defined from the resulting dendrogram using a tree height cutoff.

### Cross validation scenarios and model evaluation

Our study aims to explore how phenomics can be integrated as a selection tool in sparse testing frameworks, i.e., using image-based data from a subset of individuals to improve predictions for the entire population under selection. To simulate different sparse testing scenarios using our dataset, we first defined combinations of environments: (i) Helgegården 2020, Helgegården 2021, and Mosslunda 2021; (ii) Helgegården 2020 and Helgegården 2021; (iii) Helgegården 2020 and Mosslunda 2021; and (iv) Helgegården 2021 and Mosslunda 2021. For each combination, we applied different percentages of individuals with phenotypic records per environment, considering both conventional and image-based traits (Supplementary Fig. S1).

For combination (i), which includes three environments, we considered the following scenarios: (a) each individual was evaluated in two environments and missing in the third ($\sim$66.6% data completeness per environment); (b) each individual was evaluated in one environment and missing in the other two ($\sim$33.3% completeness); (c) based on scenario (b), some individuals lacked data in all three environments ($\sim$20% completeness); and (d) as in (c), but with only $\sim$10% data completeness per environment. For combinations (ii), (iii), and (iv), involving only two environments, we applied the following scenarios: (a) each individual was evaluated in one environment ($\sim$50% completeness per environment); (b) in addition to the missing values in (a), some individuals were missing in both environments ($\sim$40% completeness); (c) same as (b) but with $\sim$30% completeness; (d) same as (b) but with $\sim$20% completeness; and (e) same as (b) but with $\sim$10% completeness.

Each scenario was generated using R v4.1.2^23^ and repeated 50 times. For every replicate, we evaluated the performance of regression models in predicting the missing values using the following metrics: squared Pearson correlation coefficient $r^2$, Kendall’s Tau $\tau$, mean squared error (MSE), and mean absolute error (MAE).

### Prediction models

The prediction models evaluated in our study were based on linear mixed-effects models incorporating different kernel matrices to estimate the variance-covariance structures associated with genotypes, environments, and their interaction. The full model employed was:

6 $$\begin{aligned} \boldsymbol{y}=\boldsymbol{1}_{n\times m}\mu +\boldsymbol{Z_1}\boldsymbol{e}+\boldsymbol{Z_2g}+\boldsymbol{Z_3ge}+\boldsymbol{\epsilon }, \end{aligned}$$

where $\boldsymbol{y}$ is the vector of BLUEs for the conventional traits, with $n\times m$ rows (*n* genotypes evaluated across *m* environments) and one column; $\boldsymbol{1}_{n\times m}$ is a vector of ones of dimension $(n\times m)\times 1$; $\mu$ is the fixed overall population mean; $\boldsymbol{Z_1}$ is the incidence matrix linking each observation to its respective environment ($(n\times m)\times m$); $\boldsymbol{e}\sim \textrm{MVN}(0,\boldsymbol{K_1}\sigma _e^2)$ is the vector of random environmental effects; $\boldsymbol{Z_2}$ links each observation to its genotype ($(n\times m)\times n$); $\boldsymbol{g}\sim \textrm{MVN}(0,\boldsymbol{K_2}\sigma _g^2)$ is the vector of random genotype effects; $\boldsymbol{Z_3}$ links each observation to the genotype-by-environment combination ($(n\times m)\times (n\times m)$); $\boldsymbol{ge}\sim \textrm{MVN}(0,\boldsymbol{K_3}\sigma _{ge}^2)$ is the vector of genotype-by-environment interaction effects; and $\boldsymbol{\epsilon }\sim \textrm{MVN}(0,\boldsymbol{I}\sigma _\epsilon ^2)$ is the vector of residuals, assumed independent and identically distributed.

The kernel matrices considered were: (i) $\boldsymbol{K_1}$ for environments, with two alternatives, the identity matrix $\boldsymbol{I}_m$ and an environmental association matrix $\boldsymbol{E}$ (described below); (ii) $\boldsymbol{K_2}$ for genotypes, with two alternatives: the genomic relationship matrix $\boldsymbol{G}$ and identity matrix $\boldsymbol{I}_n$; and (iii) $\boldsymbol{K_3}$ for genotype-by-environment interactions, constructed as the Kronecker product $\boldsymbol{K_3}=\boldsymbol{K_1}\otimes \boldsymbol{K_2}$.

Based on the choices for $\boldsymbol{K_1}$ and $\boldsymbol{K_2}$, we considered three full-model scenarios: (i) $\boldsymbol{K_1}=\boldsymbol{I}_m$, $\boldsymbol{K_2}=\boldsymbol{G}$, $\boldsymbol{K_3}=\boldsymbol{I}_m\otimes \boldsymbol{G}$; (ii) $\boldsymbol{K_1}=\boldsymbol{E}$, $\boldsymbol{K_2}=\boldsymbol{I}_n$, $\boldsymbol{K_3}=\boldsymbol{E}\otimes \boldsymbol{I}_n$; and (iii) $\boldsymbol{K_1}=\boldsymbol{E}$, $\boldsymbol{K_2}=\boldsymbol{G}$, $\boldsymbol{K_3}=\boldsymbol{E}\otimes \boldsymbol{G}$. In each case, the response variable was modeled as:

7 $$\begin{aligned} \boldsymbol{y}\sim \textrm{MVN}\left( \boldsymbol{1}_{n\times m},\boldsymbol{Z_1}\boldsymbol{K_1}\sigma _e^2\boldsymbol{Z_1^T}+\boldsymbol{Z_2}\boldsymbol{K_2}\sigma _g^2\boldsymbol{Z_2^T}+\boldsymbol{Z_3}\boldsymbol{K_3}\sigma _{ge}^2\boldsymbol{Z_3^T}\right) . \end{aligned}$$

We also considered reduced models excluding the interaction term, with two additional scenarios: (i) $\boldsymbol{K_1}=\boldsymbol{I}_m$, $\boldsymbol{K_2}=\boldsymbol{G}$; and (ii) $\boldsymbol{K_1}=\boldsymbol{E}$, $\boldsymbol{K_2}=\boldsymbol{I}_n$. For these, the response distribution was:

8 $$\begin{aligned} \boldsymbol{y}\sim \textrm{MVN}\left( \boldsymbol{1}_{n\times m}\mu ,\boldsymbol{Z_1}\boldsymbol{K_1}\sigma _e^2\boldsymbol{Z_1^T}+\boldsymbol{Z_2}\boldsymbol{K_2}\sigma _g^2\boldsymbol{Z_2^T}\right) . \end{aligned}$$

Parameter estimation was performed in a Bayesian framework using the BGLR package v1.1.0^38^, employing a Gibbs sampler with 20,000 iterations and 2,000 burn-in cycles.

**Fig. 1:**
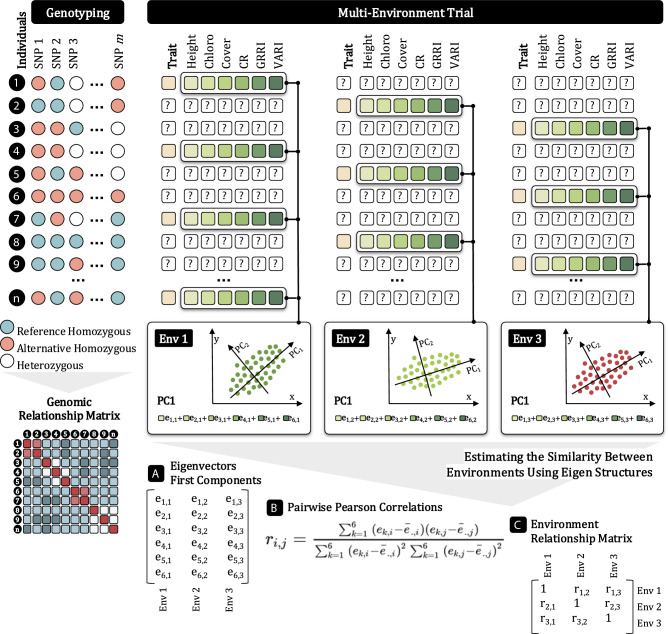
Graphical representation of the method developed for implementing sparse testing considering image-based traits in a multi-environment context, incorporating data from both conventional phenotyping and phenomics. In this scenario, individuals have phenotypic records for only a subset of environments, which prevents the direct use of these data as covariates in genomic prediction models. To address this, we performed principal component analysis (PCA) within each environment using the available data. The first principal component (PC1) eigenvectors were extracted and used to estimate pairwise Pearson correlations between environments, which were then used to define the covariance structure for modeling.

The environmental kernel $\boldsymbol{E}$ was derived from image-based traits using a PCA-based approach to overcome the challenge of sparse testing, where not all genotypes are phenotyped in all environments. The procedure (summarized in Fig. 1) involved: (i) selecting only individuals with complete image-based data for each environment; (ii) performing PCA on the image-based traits within each environment; (iii) extracting the first principal component eigenvector as a summary of each environment; and (iv) computing pairwise Pearson correlations between environments based on these eigenvectors. The resulting correlation matrix was used as the kernel $\boldsymbol{E}$.

Consider a set of *m* environments, where each environment *i* contains $n_i$ observations for six standardized image-based traits: VARI, GRRI, CR, coverage, height, and chlorophyll. For each environment *i*, let the random data matrix be denoted as $\boldsymbol{X}_i\in \boldsymbol{\mathbb {R}}^{n_i\times 6}$, with associated covariance matrix $\boldsymbol{\Sigma }_i\in \boldsymbol{\mathbb {R}}^{6\times 6}$.

Each covariance matrix $\boldsymbol{\Sigma }_i$ has 6 eigenvalues ($\lambda _{1i}\ge \lambda _{2i}\ge \lambda _{3i}\ge \lambda _{4i}\ge \lambda _{5i}\ge \lambda _{6i}$) and corresponding normalized eigenvectors $e_{1i}$, $e_{2i}$, $e_{3i}$, $e_{4i}$, $e_{5i}$, $e_{6i}$. The first principal component score for environment *i* is then defined as:

9 $$\begin{aligned} \boldsymbol{PC1}_i = e_{1i,1}\boldsymbol{X}_{i,1}+e_{1i,2}\boldsymbol{X}_{i,2}+e_{1i,3}\boldsymbol{X}_{i,3}+e_{1i,4}\boldsymbol{X}_{i,4}+e_{1i,5}\boldsymbol{X}_{i,5}+e_{1i,6}\boldsymbol{X}_{i,6} \end{aligned}$$

where $e_{1i,j}$ denotes the *j*-th component of the first eigenvector $e_{1i}$, and $\boldsymbol{X}_{i,j}$ is the vector of observations for trait *j* in environment *i*.

The first eigenvectors for all environments are assembled into a matrix:

10 $$\begin{aligned} \boldsymbol{E}_1=\left[ \boldsymbol{e}_{1,1}, \boldsymbol{e}_{1,2}, ..., \boldsymbol{e}_{1,m}\right] , \end{aligned}$$

where each column $\boldsymbol{e}_{1,i}$ represents the first eigenvector from environment *i*. Pairwise Pearson correlations are then computed between the columns of $\boldsymbol{E}_1$, and the resulting correlation coefficients define the entries of the environmental kernel $\boldsymbol{E}$.

## Results

### Phenotypic evaluation across environments

**Fig. 2:**
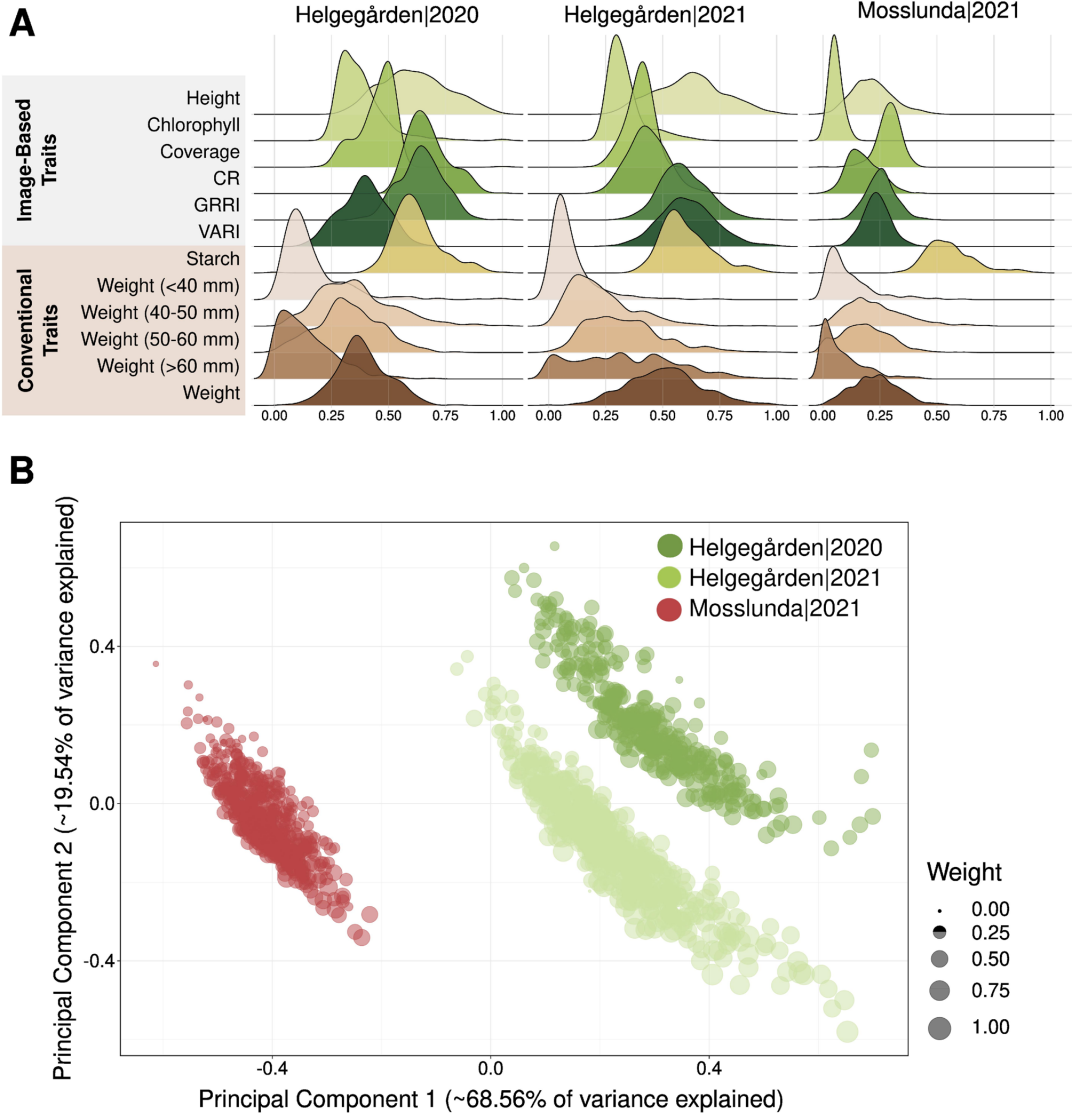
Distribution of phenotypic traits, including both conventional and image-based measurements. (**A**) Density plots of standardized phenotypic values (range 0-1) evaluated across three environments (two locations over two years). (**B**) Scatter plot of a principal component analysis (PCA) based on image-based traits. In panel B, point color represents the environment in which the measurement was taken, while point size indicates the total tuber weight (kg).

**Fig. 3:**
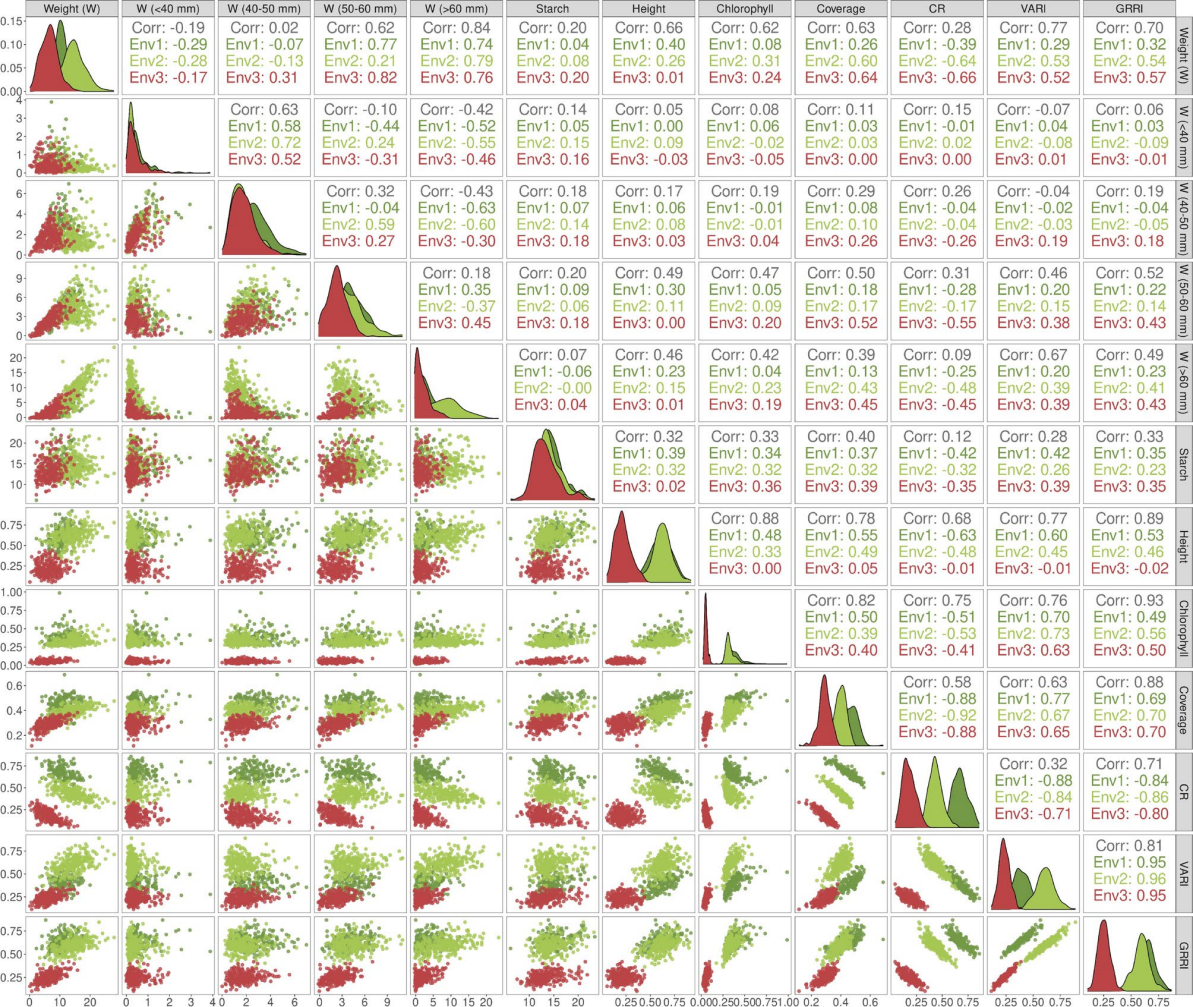
Correlation plots of best linear unbiased estimates (BLUEs) for the evaluated traits. Pairwise trait comparisons are shown separately for each environment. Values represent Pearson correlation coefficients (R), calculated both across all environments (overall) and within each environment individually. Image-based traits include: visible atmospherically resistant index (VARI), green-red ratio index (GRRI), canopy reflectance (CR), coverage, plant height, and leaf chlorophyll content. Conventional traits include: total tuber weight (W), tuber weight within defined size categories (<40 mm, 40–50 mm, 50–60 mm, and >60 mm), and tuber starch content. The environments are Helgegården|2020 (Env1), Helgegården|2021 (Env2), and Mosslunda|2021 (Env3).

We evaluated phenotypes based on both conventional and image-based traits across three environments. Density plots of raw image-based traits revealed clearer differences between environments than those observed for conventional traits (Figs. 2A and 3). For conventional traits, variation was more apparent in the shape of the distributions, suggesting differences in variance patterns. In contrast, image-based traits exhibited more distinct shifts in mean values, with an overall more consistent variance, indicating stronger environmental separation.

PCA of the image-based traits further confirmed this trend (Fig. 2B), showing clear separation among plots according to their corresponding environment. However, within each environment, the patterns of differentiation were more homogeneous, suggesting that the largest proportion of variance in image-based traits is due to environmental variation (i.e., location and year).

**Fig. 4:**
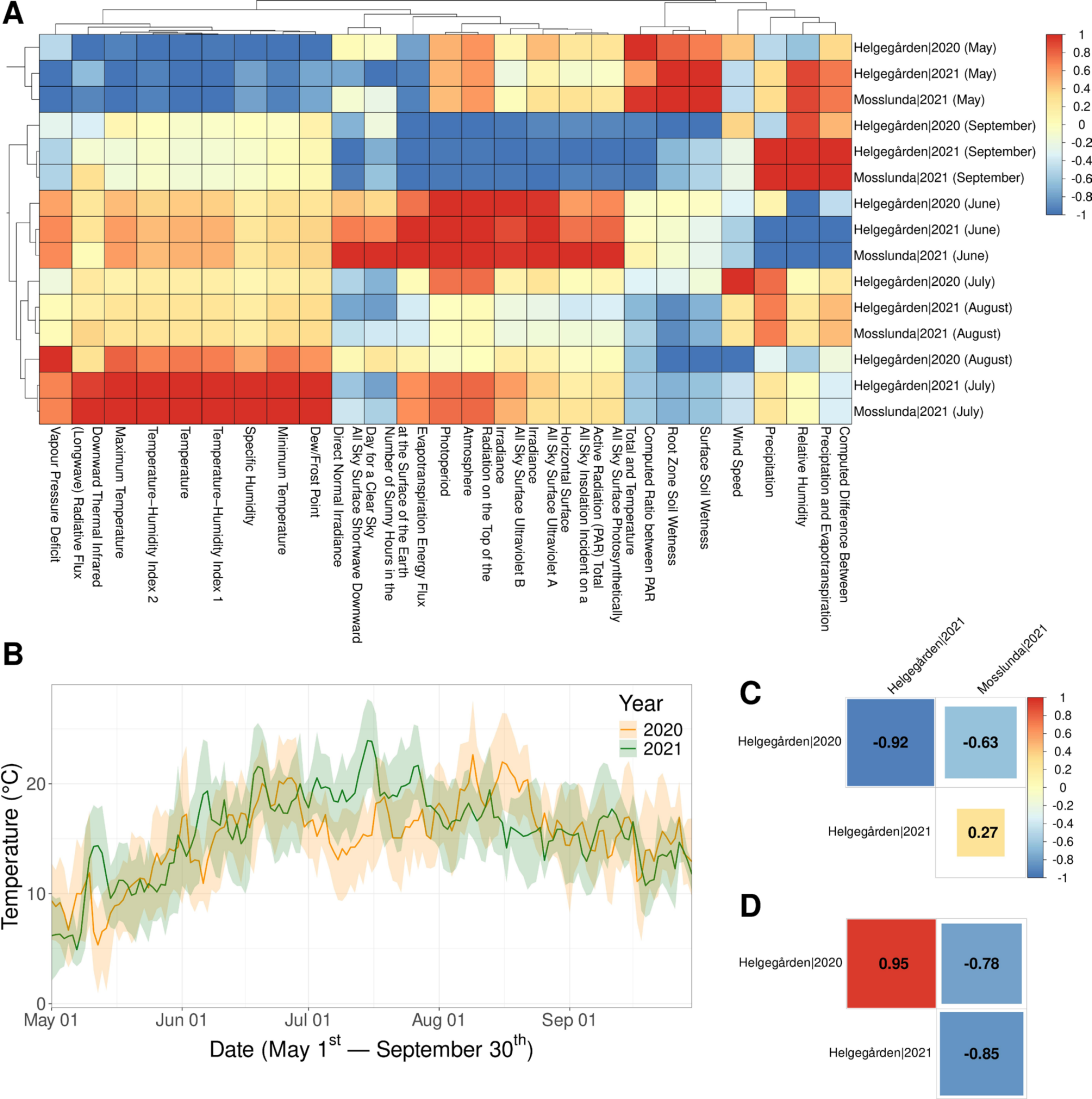
(**A**) Monthly averages of environmental covariates across the combination of two locations (Helgegården and Mosslunda) and two years (2020 and 2021) presented in a heatmap with column values standardized between -1 and 1. (**B**) Distribution of daily temperatures across the years of 2020 and 2021 in the locations employed. (**C**) Pearson correlations between environments considering the monthly averages of environmental covariates. (**D**) Pearson correlations between environments considering the eigenvector of the first principal component across principal components analyses performed in image-based traits in each environment.

Such variation was less evident in the environmental covariates measured. When comparing all environmental variables across the three environments (Fig. 4A), Helgegården|2021 and Mosslunda|2021 appeared more similar within each month, while Helgegården|2020 exhibited a distinct profile. This likely results from the geographic proximity of the locations, where temperature differences were observed between years, but not between locations (Fig. 4B). It is important to note that these observations may be affected by the limited resolution of the sensors associated with the environmental covariates used.

### Genetic analyses and trait heritabilities

Using linear mixed models, we estimated both Cullis heritability and broad-sense heritability for conventional and image-based traits. In all cases, Cullis heritability was higher than the corresponding broad-sense heritability. This likely results from reduced prediction error variance due to the experimental design employed and the presence of significant genotype-by-environment interactions (Supplementary Table S2). All traits, except plant height, showed significant genotype-by-environment and genotype main effects (p < 0.05). Plant height had near-zero heritability, while the remaining traits exhibited moderate to high heritability estimates, with conventional traits generally showing higher values (up to $\sim$0.67 for starch content).

Environmental effects were significant for all traits (p-value < 0.05). The nested effect of replicate within environment was significant only for the image-based traits (Supplementary Table S3), highlighting their sensitivity to subtle field variation. Indeed, image-based traits demonstrated high discriminative power across experimental units, with significant effects even for field layout factors such as rows and columns (Supplementary Table S2). Such patterns were less pronounced for conventional traits.

In order to evaluate the genotype trends of variance across traits, we employed the established mixed models excluding the genotype main effect, thus focusing on the genotype-by-environment effects. For most traits, the heterogeneous first-order autoregressive variance-covariance structure showed the lowest AIC and BIC values (Supplementary Table S4), indicating: (i) correlation among environments; (ii) heterogeneity of variances across environments; and (iii) a spatial/temporal trend in similarity.

Using BLUPs estimated under this model structure (Supplementary Fig. S2), we observed a consistent correlation pattern across traits: correlations between Helgegården|2020 and Helgegården|2021 were comparable to those between Helgegården|2021 and Mosslunda|2021; and correlations between Helgegården|2020 and Mosslunda|2021 were lower, reinforcing that environmental similarity follows a temporal and spatial gradient.

For prediction models, BLUEs for genotype-by-environment effects were used. Trait-to-trait correlations based on BLUEs further revealed key relationships (Fig. 3). Notably, starch showed low correlation with other traits and maintained consistent values across environments. Weight-based measurements could be grouped into two categories: (i) total weight, and weights for tubers 50–60 mm and >60 mm; and (ii) weights for tubers <40 mm and 40–50 mm. Traits within each category were more strongly correlated internally, with group (i) traits also showing higher correlations with image-based traits. For example, VARI and total weight showed a maximum correlation of 0.77.

Although BLUEs showed more similar correlations across environments (e.g., total weight had a Pearson correlation of approximately 0.64 between Helgegården|2020, Helgegården|2021, and Mosslunda|2021) (Supplementary Fig. S3), correlations between image-based and conventional traits tend to be more environment-specific. While VARI and total weight showed an overall correlation of 0.77, this value dropped when examined separately per environment, with correlations of approximately 0.29 for Helgegården|2020, 0.53 for Helgegården|2021, and 0.52 for Mosslunda|2021. These findings indicate that the relationship between conventional tuber traits and plant physiology or vegetative growth, measured via image-based traits, is specific to each environment. Therefore, trends observed under one set of environmental conditions may not hold in others (Supplementary Figs. S2–S3).

**Fig. 5:**
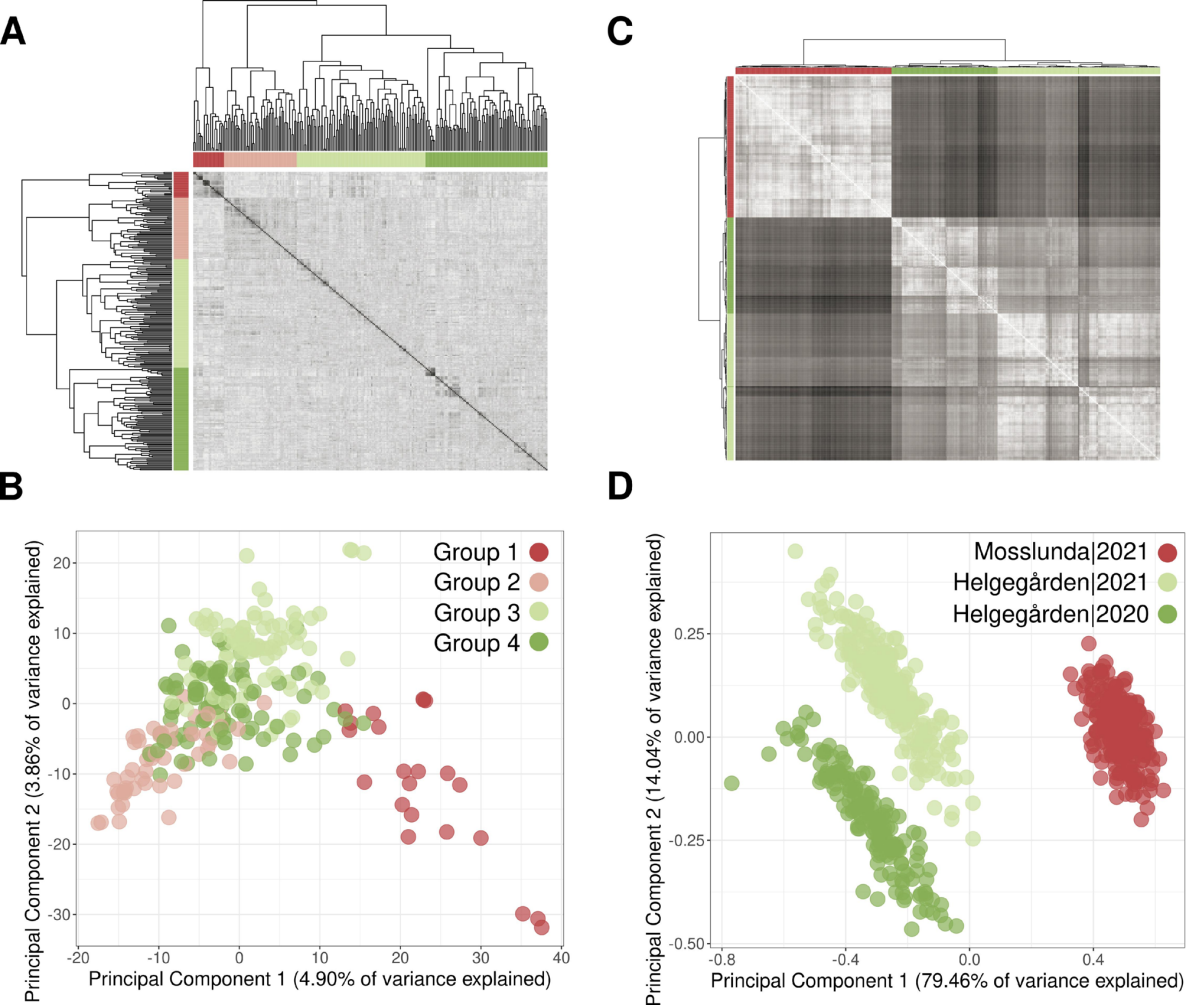
Multivariate analyses using genomic and phenomic datasets. (**A**) Heatmap of the genomic relationship matrix (GRM), with hierarchical clustering performed using Ward’s minimum variance method. (**B**) Principal component analysis (PCA) of the genomic data, where each point represents a genotype and colors indicate cluster assignments based on the dendrogram from (**A**). (**C**) Heatmap of the Euclidean distance matrix computed from best linear unbiased estimates (BLUEs) of image-based traits, clustered using the same hierarchical method as in (**A**). (**D**) PCA plot of the image-based trait data, with genotypes colored according to their corresponding environment.

PCA and the SNP-based genomic relationship matrix did not reveal strong population structure (Fig. 5A–B). Although five clusters were identified through hierarchical clustering, the separation was not clearly supported by the genomic relationship matrix. This suggests that random cross-validation strategies in genomic prediction are unlikely to be biased by population structure in this dataset. In contrast, the phenomic relationship matrix (Fig. 5C), based on BLUEs from image-derived traits, clearly separates individuals by environment. This pattern was consistent with the PCA results and the initial evaluations of raw trait distributions.

### Defining the association between environments using image-based traits

The previous results demonstrated that image-based traits effectively captured differences between environments. Moreover, these traits also captured significant variation associated with plot structure factors such as replicates, rows, and columns. By incorporating a heterogeneous first-order autoregressive variance-covariance structure into the mixed models for each trait and evaluating the correlations between BLUPs, we were able to establish a similarity pattern among environments, a pattern that was not as evident when using conventional environmental covariates (Fig. 4A).

While we acknowledge the limitations of this environmental characterization, especially for geographically close locations, our results suggest that the predictive power of these environmental covariates in our multi-environment trials is limited. Indeed, the correlations between environments based on these covariates (Fig. 4C) did not reflect the actual trait correlations observed across environments (Supplementary Figs. S2–S3).

PCAs of the raw image-based data (Fig. 2B) and the BLUEs of genotype-by-environment interactions (Fig. 5D) revealed that environmental variation accounted for the largest proportion of variance in image-based traits. To investigate whether this pattern was consistent across environments, we performed separate PCAs for each environment and computed correlations between the eigenvectors associated with their first principal components (Fig. 4D). The resulting correlation matrix exhibited magnitudes comparable to those observed among the BLUPs of genotype-by-environment effects. Notably, the correlations involving Mosslunda|2021 were negative relative to other environments, likely reflecting the distinct management at that site.

Given that the correlation between the first eigenvectors of each environment provided a reasonable measure of similarity among environments, we explored the potential of using this matrix as an environmental kernel in multi-environment prediction models. However, it is important to emphasize that for other crops or experimental setups involving different environments and management practices, this structure may not be directly transferable, and alternative components may need to be considered.

### Prediction models for sparse testing across three environments

The cross validation scenarios employed in this study were designed to represent sparse testing scenarios. Initial evaluations of the prediction models were done under all three environments, with varying proportions of complete data per environment (Supplementary Table S5). For all traits, including a genotype-by-environment interaction term produced results comparable to models without it when 66% of the data were available per environment. Even in this more complete setting, differences were minimal and inconsistent across evaluation metrics. In scenarios with higher proportions of missing data, model performance decreased when the interaction term was included, likely due to the limited number of individuals available for reliable estimation. Consequently, we focused on models excluding the genotype-by-environment interaction term.

In highly sparse scenarios, with only 10% or 20% complete information per environment, mean $r^2$ values across cross-validation replicates were below 0.5 for all traits. The highest $r^2$ (approximately 0.47, corresponding to a Pearson correlation coefficient of $\sim$0.7) was observed for starch content at 20% data availability using the $\boldsymbol{G}$ kernel. This finding aligns with the high inter-environment correlations observed for starch BLUEs (Supplementary Fig. S3). However, under these conditions of high missing data, the environmental kernel $\boldsymbol{E}$ did not outperform the $\boldsymbol{G}$ kernel.

**Fig. 6:**
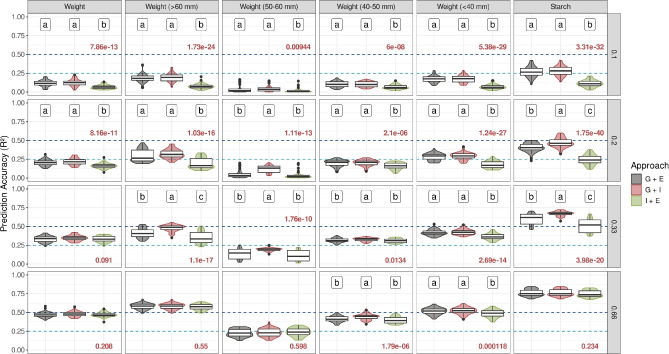
Prediction accuracy (squared Pearson correlation coefficients) of regression models using different kernel combinations for predicting the best linear unbiased estimates (BLUEs) of conventional traits. For genotype-related kernels, either the genomic relationship matrix (G) or the identity matrix (I) was used. For environment-related kernels, options included the environmental relationship matrix derived from principal component analysis (E) or the identity matrix (I). The prediction strategy followed the CV2 cross-validation scheme across three environments, considering varying proportions of complete data per environment. ANOVA was used to test for significant differences among models for each trait–data proportion scenario, with p-values shown in red. The Scott-Knott procedure identified the top-performing models, indicated by labels adjacent to the corresponding boxplots.

At 33% data availability, starch remained the only trait with $r^2$ values exceeding 0.5. For tuber weight in the largest size class (>60 mm), the $\boldsymbol{G}$ kernel achieved an average $r^2$ of $\sim$0.48 ± 0.04 across cross-validation replicates, while the $\boldsymbol{E}$ kernel yielded a lower average $r^2$ of $\sim$0.35 ± 0.08. However, when evaluating prediction similarity using alternative metrics, the differences were less pronounced ($\tau$ = 0.52 ± 0.03 for $\boldsymbol{G}$ and $\tau$ = 0.44 ± 0.05 for $\boldsymbol{E}$). For total weight, no statistically significant differences were detected between the $\boldsymbol{G}$ and $\boldsymbol{E}$ kernels (Fig. 6). Although the $\boldsymbol{G}$ kernel generally delivered higher predictive ability across traits, the $\boldsymbol{E}$ kernel showed competitive performance, especially when considering alternative metrics such as $\tau$, MSE, and MAE.

When 66% of the data were available, the kernel $\boldsymbol{G}$ and $\boldsymbol{E}$ performed equally well for starch content, total tuber weight, and weight of large tubers in the 50-60 mm and >60 mm categories. For tuber weight in the smaller size classes (<40 mm and 40-50 mm), the $\boldsymbol{G}$ kernel performed better, although the $\boldsymbol{E}$ kernel still provided comparable results. Mean accuracies were above 0.5 for most traits, except for total tuber weight and weight in the 40-50 mm and 50-60 mm size classes. Notably, combining the $\boldsymbol{G}$ and $\boldsymbol{E}$ kernels did not result in improved predictive accuracy under any scenario evaluated.

### Prediction models for sparse testing across two environments

When considering pairwise combinations of environments, a different trend emerged. For all traits evaluated with 50% complete data per environment, the exclusive use of the $\boldsymbol{E}$ kernel outperformed both the $\boldsymbol{G}$ kernel and the combined $\boldsymbol{G}+\boldsymbol{E}$ model (Supplementary Table S6). For starch, the gains were modest due to the already high predictive accuracy with the $\boldsymbol{G}$ kernel. However, for other traits, the improvements were more substantial, including up to $\sim$62% for tuber weight in the 50–60 mm category (Helgegården 2020 and Mosslunda 2021), and $\sim$60% for total tuber weight (Helgegården 2021 and Mosslunda 2021). Across all traits and environment combinations at 50% data availability, the exclusive use of $\boldsymbol{E}$ kernel significantly outperformed the $\boldsymbol{G}$ kernel and the combined kernel approach (Supplementary Figs. S4–S6).

At 40% data availability, the $\boldsymbol{E}$ kernel outperformed the $\boldsymbol{G}$ kernel for total tuber weight and for tuber weight in the 40-50 mm and 50-60 mm size classes. For the smallest (<40 mm) and largest (>60 mm) tuber size categories, no significant differences were observed in most comparisons. At 30% data availability, performance varied depending on the specific environment combinations. Under the most extreme sparsity levels (10% and 20%), no consistent trend emerged; however, the $\boldsymbol{G}$ kernel generally performed better in most scenarios.

The prediction accuracies observed for the proposed approach are notably high (Table 2). Across all scenarios with at least 33% complete data per environment, the environmental kernel ($\boldsymbol{E}$) either matched or outperformed the genomic kernel ($\boldsymbol{G}$), suggesting that $\boldsymbol{E}$ can serve as an effective substitute for $\boldsymbol{G}$ in sparse phenotyping contexts.

**Table 2:** Prediction accuracy (expressed as squared Pearson correlation coefficients) of regression models using different kernel combinations for predicting the best linear unbiased estimates (BLUEs) of total tuber weight across sparse testing. Genotype-related kernels included either the genomic relationship matrix ($\boldsymbol{G}$) or the identity matrix ($\boldsymbol{I}$), while environment-related kernels included either the environmental relationship matrix derived from principal component analysis ($\boldsymbol{E}$) or the identity matrix ($\boldsymbol{I}$). Results are presented as the mean ± standard deviation across replicates. In scenario 1, 66% of complete data is considered in three environments and 50% in two environments. In scenario 2, 33% of complete data is considered in three environments and 40% in two environments.

Environment combination	Kernel	Scenario 1	Scenario 2
Helgegården (2020 and 2021) and Mosslunda (2021)	$\boldsymbol{G}$ + $\boldsymbol{I}$	0.481 ± 0.035	0.347 ± 0.035
	$\boldsymbol{I}$ + $\boldsymbol{E}$	0.472 ± 0.031	0.332 ± 0.040
	$\boldsymbol{G}$ + $\boldsymbol{E}$	0.470 ± 0.035	0.333 ± 0.041
Helgegården (2020) and Mosslunda (2021)	$\boldsymbol{G}$ + $\boldsymbol{I}$	0.798 ± 0.005	0.568 ± 0.051
	$\boldsymbol{I}$ + $\boldsymbol{E}$	0.996 ± 0.007	0.662 ± 0.064
	$\boldsymbol{G}$ + $\boldsymbol{E}$	0.798 ± 0.008	0.567 ± 0.051
Helgegården (2021) and Mosslunda (2021)	$\boldsymbol{G}$ + $\boldsymbol{I}$	0.612 ± 0.006	0.440 ± 0.037
	$\boldsymbol{I}$ + $\boldsymbol{E}$	0.997 ± 0.007	0.668 ± 0.056
	$\boldsymbol{G}$ + $\boldsymbol{I}$	0.611 ± 0.007	0.440 ± 0.038
Helgegården (2020 and 2021)	$\boldsymbol{G}$ + $\boldsymbol{I}$	0.787 ± 0.004	0.591 ± 0.040
	$\boldsymbol{I}$ + $\boldsymbol{E}$	0.995 ± 0.009	0.676 ± 0.058
	$\boldsymbol{G}$ + $\boldsymbol{E}$	0.786 ± 0.007	0.591 ± 0.041

## Discussion

The advent of new HTP technologies has enabled rapid, cost-effective, and non-destructive monitoring of plant growth^12^. These technologies generate phenomic markers with strong potential as complementary indicators for a wide range of traits^39^. However, the utility of HTP in predictive breeding for complex traits has been debated. This is largely due to the observation that associations between conventional and image-based traits vary depending on the environment and developmental stage^11^. In our study, instead of using HTP data to directly predict conventional traits, we employed image-derived information to characterize environmental variation. This approach leverages the sensitivity of image data to capture differences not only across environments but also within experimental units such as replicates, columns, and rows.

We propose that this strategy represents a novel way to incorporate image-based data into sparse testing designs, an approach that has shown great promise for increasing the number of genotypes evaluated under fixed resource constraints^10^. The main challenge of incorporating image-based data in sparse testing is how to deal with individuals that have not been phenotyped in the field. Some studies have addressed this by employing multi-trait models, treating image-based traits as secondary variables^40,41^. However, this method becomes less practical when the number of digital traits increases. Alternatively, using a regression kernel based on image traits is an effective strategy for handling high-dimensional data^42^, though it may be impractical in sparse testing scenarios. In this context, we propose the use of an environmental kernel derived from HTP data as a solution to these challenges. Such a kernel can reveal patterns of environmental similarity and improve prediction models.

PCA is a well-established multivariate technique in which linear combinations of original features produce new orthogonal variables, known as principal components^43^. These components are derived from the eigenvectors of the covariance matrix and are ranked by the magnitude of their corresponding eigenvalues, each representing a specific proportion of the total variance in the dataset. In our study, we conducted separate PCAs on the image-based traits for each environment, using the first principal component as a representative measure of the main morphological and physiological variation.

Given that the first component captures the largest proportion of variance of a dataset (approximately 80% in the case of the BLUEs of image-based traits, Fig. 5D), we considered it an effective summary of the major phenotypic trends within each environment. Subsequent components were excluded because the remaining variance may partition differently across environments, which would complicate pairwise comparisons. Furthermore, because each component explains a different proportion of variance depending on the environment, incorporating them would require complex distance-weighting adjustments. Accordingly, we extracted the eigenvector associated with the first principal component from each PCA to characterize the corresponding environment. These vectors were then used as inputs to construct the environmental kernel, enabling the integration of environment-specific variation into our subsequent analyses.

Although there is still considerable ongoing research^44^, especially on crops presenting additional challenges, one of the key lessons plant breeders have learned regarding GS is that predicting the performance of new lines in untested environments remains a significant challenge, particularly in light of increasing climate variability^2^. The use of environmental covariates to quantify environmental differences is already a well-established strategy in predictive breeding^45^. However, the effectiveness of this approach depends on the quality and relevance of sensor-based data^18^. In environments with limited sensor information, spatial proximity, or unclear environmental patterns, this method is less effective, as observed in our study. Conversely, our proposed approach performs well in such conditions, particularly in micro-environments with subtle differences. In trials involving different management practices, for example, HTP data clearly captures plant responses to various stressors^46^. In these cases, environmental kernels derived from image data can provide even more informative distinctions than those based solely on weather variables.

In potato breeding, GS prediction accuracies for highly quantitative traits remain relatively low^47,48^. Although GS has the potential to reduce the need for extensive field trials, the expected gains in selection accuracy do not always translate into substantial improvements in genetic gain. Adjusting the composition of the training population can improve predictive performance^7^, but predictions in untested environments remain a challenge. Nevertheless, genome-enabled sparse testing has shown promise in enhancing genomic prediction accuracy in potatoes^33^.

In our study, we observed encouraging results across several evaluated scenarios and traits. Despite the use of a relatively limited number of SNP markers, previous work^32^ has demonstrated that such marker density is sufficient to produce reliable estimates in potato, which aligns with our findings. Nevertheless, we acknowledge that the relatively limited sample size and the small number of evaluated environments may have influenced model performance.

Consistent with earlier studies^12,49^, our results also underscore the value of phenomics in monitoring crop development. Distinct phenotypic differences across environments were clearly captured (Figs. 2 and 3), supporting the utility of image-derived traits for environmental characterization. Notably, in certain scenarios the predictive performance of the environmental kernel built from image-based traits was comparable to that of the genomic relationship matrix. In our experimental conditions, where environments were relatively similar, HTP-derived information may better capture subtle environmental variation than traditional environmental covariates.

Although correlations of BLUEs across environments were variable among traits, positive results were observed even for traits with comparatively lower correlations, such as tuber weight by size category. Furthermore, genomic prediction in potato is often challenging due to the complex genetic architecture of the crop, which may reduce the ability of the genomic relationship matrix to fully capture genotype-specific responses. Consequently, part of the predictive contribution of the genomic kernel may effectively behave similarly to an identity component, potentially explaining the limited additional gains observed when including both terms in some scenarios (or even the G$\times$E term).

While genomic data provided more robust predictions in extreme cases with minimal missing data, in all situations where at least 30% of individuals per environment had complete data, predictions of total tuber weight using the image-based environmental kernel were as accurate, or in some cases more accurate, than those using genomic data alone.

Predictive performance was generally higher in the two-environment scenarios compared with the three-environment scenarios. We believe this result is associated with the increase in the covariance components that must be estimated as additional environments are incorporated into the model. Given the limited dataset size employed in our study, models with fewer environments were likely easier to parameterize, leading to improved predictive ability. In addition, although increasing the number of environments generally increases the amount of available data, this advantage may not fully compensate for the increased model complexity when the total number of observations remains limited. The same reasoning may explain the relatively small contribution of the G$\times$E term, whose estimation likely requires larger datasets and more diverse environmental conditions to provide substantial predictive gains.

We also acknowledge two additional limitations. First, because the environmental kernels were derived from image-based phenotypes collected within the same experimental framework used for model training and validation, some degree of information leakage may be present in the G+E models. Second, the evaluated environments were relatively similar, which limits the generalization of our results to more contrasting conditions or other crops. Future studies should therefore include stricter validation schemes and independent environments with stronger contrasts to better assess the robustness and transferability of image-based environmental kernels. In addition, the balance between the number of individuals and the number of environments should be further investigated to optimize predictive performance in multi-environment genomic prediction models.

Given that the implementation of GS may not yet be cost-effective for some small-scale breeding programs, our findings highlight the potential of integrating an environmental kernel derived from phenomic data into sparse testing schemes. In some cases, this approach may even serve as a viable alternative to genomic information. For programs that already have access to genomic data, combining it with phenomic-based environmental characterization can further enhance predictive accuracy.

Image-based phenomics can be used in early selection, when a large number of genotypes are evaluated across diverse environments, producing more accurate estimates of genetic values. This could reduce the number of replicated genotypes while expanding the total number of genotypes evaluated. In later stages, genomic data can then be applied to a smaller, refined subset of individuals to yield more precise estimates, complementing the phenomic information. Furthermore, breeding programs that already use image-based crop monitoring systems could reuse this data for predictive modeling at no additional cost.

Given that potato is highly sensitive to various biotic and abiotic stressors^15^, UAV-based phenotyping provides valuable, non-invasive indicators of plant physiological status^50^. Previous studies have shown that image-derived traits can also support disease detection and prediction in potatoes^51–54^. Thus, the integration of phenomics into breeding workflows is both practical and cost-effective, making it an attractive strategy for breeding programs of varying scales. Moreover, considering the long breeding cycles in potato, such approaches may contribute to reducing the number of genotypes evaluated per environment or year without compromising genetic gain.

Among the traits analyzed, total tuber weight, an indicator of yield, showed the most promising prediction results. Although breeding priorities vary, yield remains a key selection target in virtually all potato breeding programs^15^. On the other hand, for nutritional traits such as starch content, environmental differences are less pronounced. As a result, the environmental kernel derived from image-based traits did not substantially improve prediction accuracy for these traits. However, other HTP techniques may provide more precise information for such traits, for example, near-infrared spectroscopy (NIRS)^55^. More research is clearly needed to assess the potential of phenomics across different trait categories and breeding objectives.

## Supplementary Information

Supplementary Information 1.

Supplementary Information 2.

## Data Availability

The marker and phenotypic data used in this study are available in the supplementary materials accompanying the manuscript (Supplementary Tables S7-S8).
