# Supplementary material for: Phenomics-assisted sparse testing for potato breeding
Source: Sci Rep. 2026 Jul 14;16:22033. doi: 10.1038/s41598-026-59202-6 (PMC13365407; doi:10.1038/s41598-026-59202-6)
Supplement: Supplementary file 1 — Supplementary Information 1. [file 41598_2026_59202_MOESM1_ESM.pdf]

Phenomics-Assisted Sparse Testing for Potato Breeding

Supplementary Figures

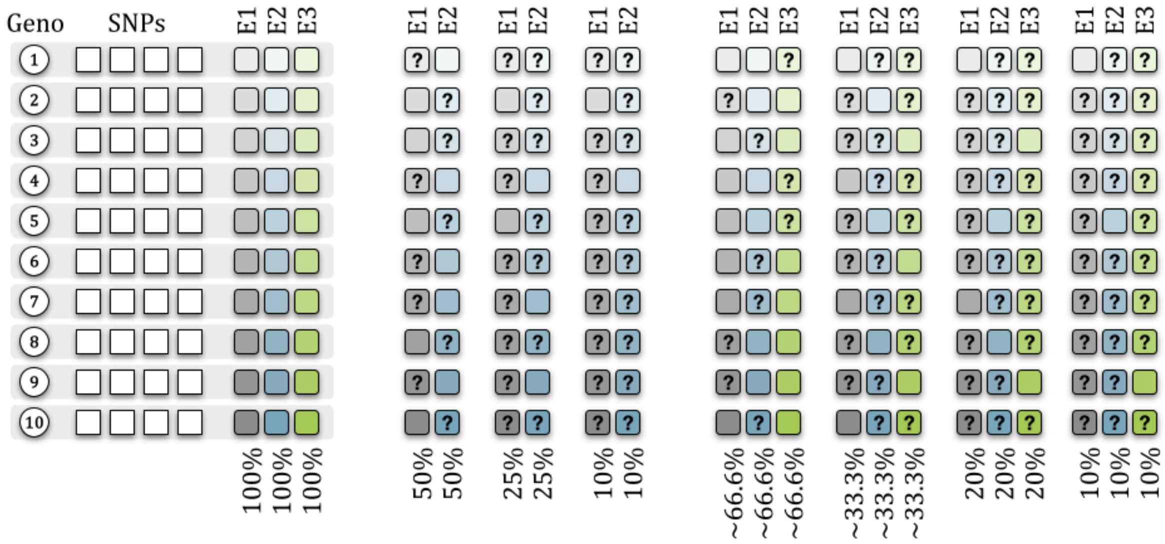

Supplementary Fig. S1. Graphical representation of the sparse testing or cross-validation scheme 2 (CV2) applied in multi-environment genomic selection. The dataset is partitioned into training and testing sets by randomly splitting individuals across different environments.

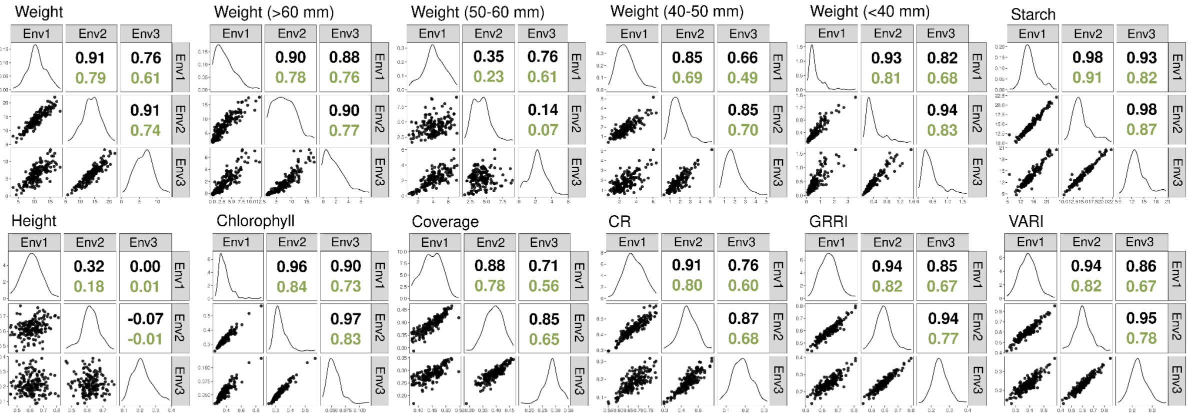

Supplementary Fig. S2. Correlation plots of best linear unbiased predictions (BLUPs) for each trait across the three environments, based on the genotype-by-environment interaction term modeled with a heterogeneous first-order autoregressive variance-covariance structure. For each trait, pairwise comparisons between environments are shown. The first value (black) represents the Pearson correlation coefficient, and the second value (green) indicates the Kendall rank correlation coefficient. The environments are Helgegården|2020 (Env1), Helgegården|2021 (Env2), and Mosslunda|2021 (Env3).

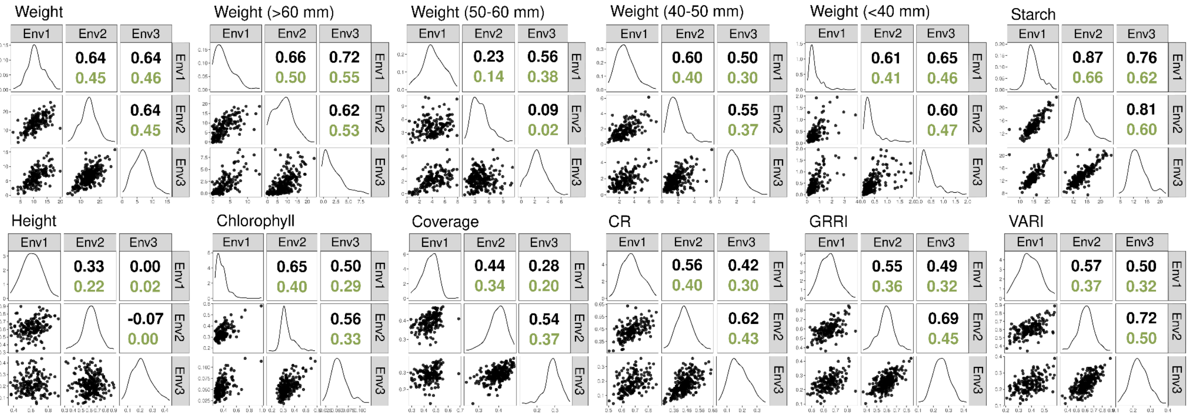

Supplementary Fig. S3. Correlation plots of best linear unbiased estimates (BLUEs) for each trait across the three environments, based on the genotype-by-environment interaction term. For each trait, pairwise comparisons between environments are shown. The first value (black) represents the Pearson correlation coefficient, and the second value (green) indicates the Kendall rank correlation coefficient. The environments are Helgegården|2020 (Env1), Helgegården|2021 (Env2), and Mosslunda|2021 (Env3).

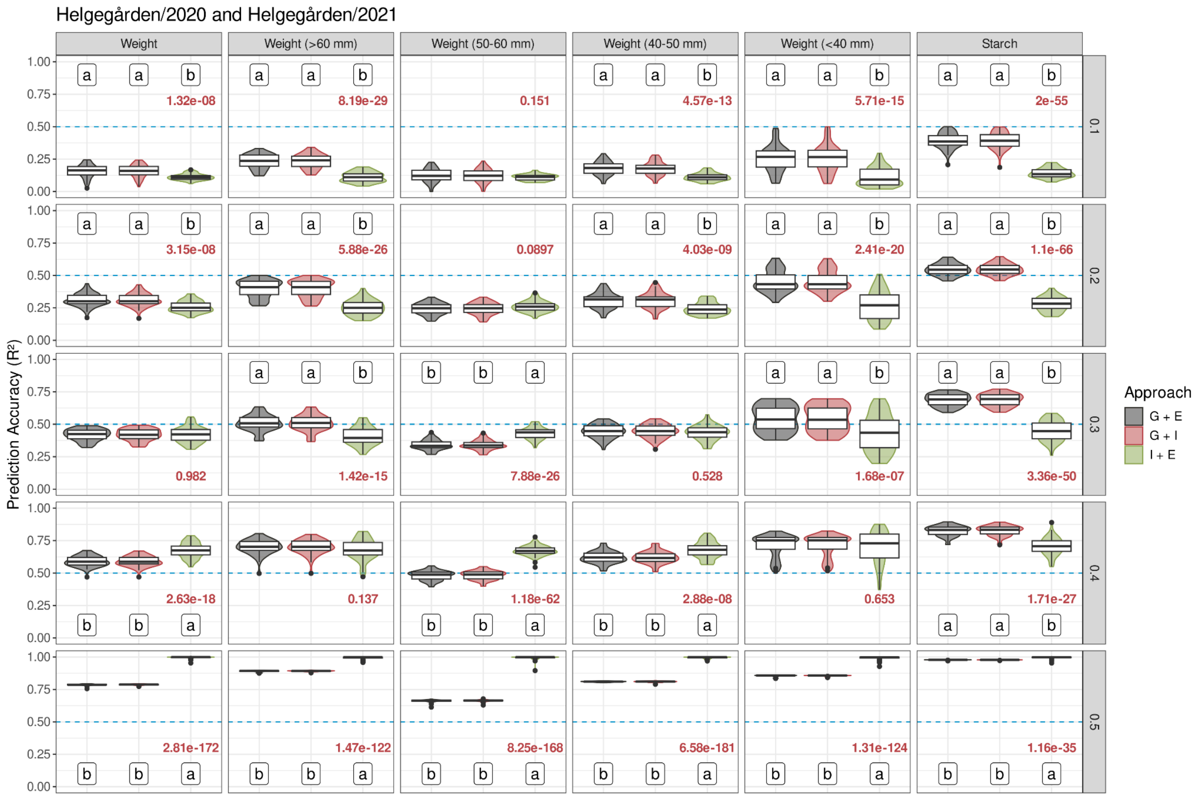

Supplementary Fig. S4. Prediction accuracy (squared Pearson correlation coefficients) of regression models using different kernel combinations for predicting the best linear unbiased estimates (BLUEs) of conventional traits. For genotype-related kernels, either the genomic relationship matrix (G) or the identity matrix (I) was used. For environment-related kernels, options included the environmental relationship matrix derived from principal component analysis (E) or the identity matrix (I). The prediction strategy followed the CV2 cross-validation scheme across Helgegården 2020 and Helgegården 2021, considering varying proportions of complete data per environment. ANOVA was used to test for significant differences among models for each trait–data proportion scenario, with p-values shown in red. The Scott-Knott procedure identified the top-performing models, indicated by labels adjacent to the corresponding boxplots.

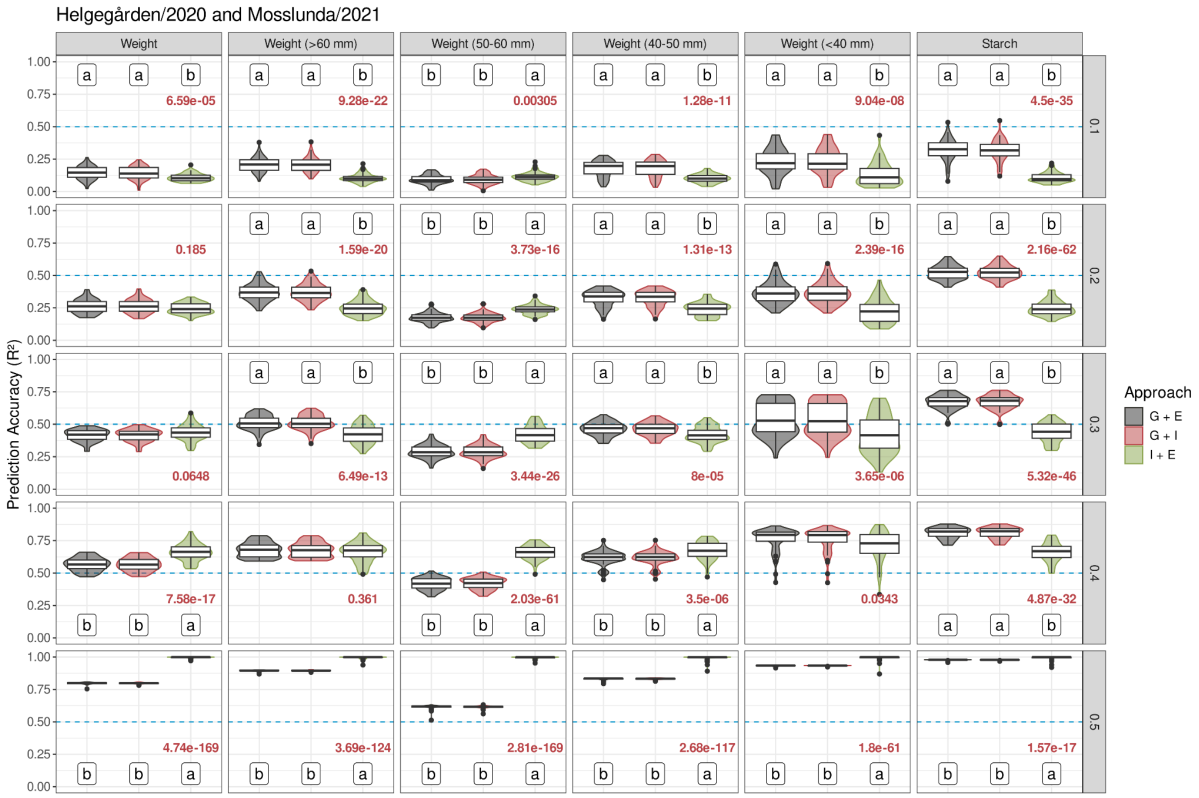

Supplementary Fig. S5. Prediction accuracy (squared Pearson correlation coefficients) of regression models using different kernel combinations for predicting the best linear unbiased estimates (BLUEs) of conventional traits. For genotype-related kernels, either the genomic relationship matrix (G) or the identity matrix (I) was used. For environment-related kernels, options included the environmental relationship matrix derived from principal component analysis (E) or the identity matrix (I). The prediction strategy followed the CV2 cross-validation scheme across Helgegården 2020 and Mosslunda 2021, considering varying proportions of complete data per environment. ANOVA was used to test for significant differences among models for each trait–data proportion scenario, with p-values shown in red. The Scott-Knott procedure identified the top-performing models, indicated by labels adjacent to the corresponding boxplots.

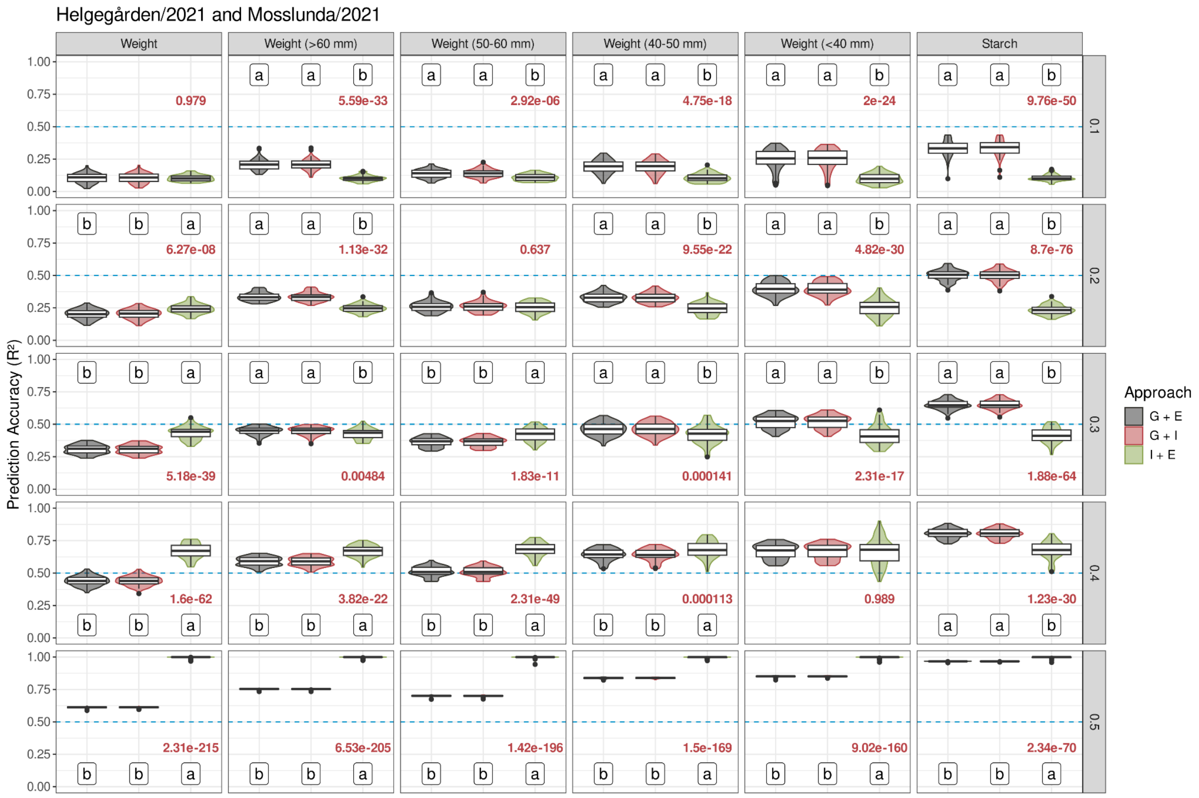

Supplementary Fig. S6. Prediction accuracy (squared Pearson correlation coefficients) of regression models using different kernel combinations for predicting the best linear unbiased estimates (BLUEs) of conventional traits. For genotype-related kernels, either the genomic relationship matrix (G) or the identity matrix (I) was used. For environment-related kernels, options included the environmental relationship matrix derived from principal component analysis (E) or the identity matrix (I). The prediction strategy followed the CV2 cross-validation scheme across Helgegården 2021 and Mosslunda 2021, considering varying proportions of complete data per environment. ANOVA was used to test for significant differences among models for each trait–data proportion scenario, with p-values shown in red. The Scott-Knott procedure identified the top-performing models, indicated by labels adjacent to the corresponding boxplots.
